# Practice vs. Theory

**Published:** 1867-08

**Authors:** 


### EDITORIAL DEPARTMENT.

*Practice vs. Theory.*—Few men have thrown greater obstacles in the way of scientific progress than those who are perpetually vaunting themselves to be exclusively practical. The narrow horizon of their individual experience shuts them in, and they cannot be brought to believe that beyond the limits of their native valley, and out of sight of their own chimneys' smoke, there is a great world full of mighty energies and prodigious works. Because they cannot see the immediate bearing of scientific research upon their daily life and its ever recurring labours, they disregard and deride all disinterested inquiry into truth. How often do we hear the cry "*cui bono*" raised in reference to mental labours, and how often in the progress of the world has its absurdity been shown.

The truth is that, in many respects, the exclusively practical man is singularly unpractical. He has a set of rules by which he works, without knowing anything of the foundation on which they rest. Thus he very often is guided by the worn-out fallacies of old speculations. A superstitious adherence to formulæ, he imagines to be practical independence. How many practical farmers of the present day consult the almanac to know the proper sign in which to sow their wheat, or plant their turnips. How many more have wise saws about the quarters of the moon in which to kill their meat or set their potatoes. And how astounded and incredulous they are when told that these formulæ which they imagine to be practical rules, are in truth the merest dreams of the oldest and most extravagant theorizers in the world, the astrologers of Chaldea;—vain imaginations which have long been set aside by men of science. So too it would be easy to show that many of the popular notions of medicine are derived from exploded theories, such as the old humoural pathology, or the doctrine of signatures, or some other equally wild and absurd speculation. The truth is that the general public attires itself in the cast-off garments of science, and is usually a century or two behind the times.

Now we do not mean to deny the value of experience, provided it be real and not imaginary. Unfortunately, however, much that claims to be experience is really made up of a very few imperfectly observed facts, mixed with a vast amount of utterly false reasoning upon those facts, and much superstition, often descending from a remote antiquity. True experience always goes hand in hand with science. Indeed, science, in the proper acceptation of the term, is nothing else than the orderly arrangement of the world's experience.

It is no answer to such statements to say that art is always in advance of science. In the first place, the statement is true only to a limited extent and for a limited period of the history of art. The first efforts both of science and art are tentative. Art searches out the secrets of nature, feels after them as it were. It tries first one method then another, and after a variety of blind endeavours, finally hits upon a desirable combination. All that the artist knows, however, is that his process works well. The moment he leaves his empirical formulæ and begins to inquire into the reason of success or failure, he trenches upon the domain of science. It is here that science begins. Arranging the materials provided for her by her elder sister, she inquires into cause and effect, and discovers the shortcomings of art. Criticism becomes possible, and gives light which mere empiricism never could evolve.

To illustrate from a familiar art, that of the dyer. For a long time it was a mere collection of formulæ, and each man worked by a routine marked out by his predecessors. In these latter days, however, chemistry has begun to study the substances employed by the dyer. The action of mordants is now understood, the changes affected upon the dye-stuffs by different chemical reactions have been made known. Wider research has extended the knowledge of the tinctorial resources of nature, and refuse material hitherto useless, has been made to yield tints of unsurpassed delicacy and splendor. The whole art has been revolutionized and reached a degree of perfection which never could have been obtained without the aid of science.

It is clear then, that while art has its special functions to perform, and must always precede science, it can never reach its full perfection without the aid of the latter. How idle then to object, as many do, to the minute inquiry into all the phenomena of the world. How absurd the perpetually repeated cry of *cui bono!* How ridiculous to the practical man, the man of formulæ and routine, must have appeared the chemists' researches into the combinations of the radical formyl. Yet out of these inquiries came chloroform, the greatest boon of modern surgery to suffering humanity.

Such results should caution people to beware how they condemn hastily inquiries of which they do not see the immediate practical bearing. Every truth is valuable because it is truth, and all the phenomena of the universe should be carefully studied. Truth is precious even in its fragments, and he who begins by despising it in its humble manifestations, will end by scoffing at its loftiest developements. The new fact may be neglected at first, may be overlaid with rubbish, sunk out of sight, forgotten, but the perennial vitality of truth is in it, and it must arise out out of its ashes, vigorous and glorious. So the acorn is trodden under foot, hidden in leaves, buried in the soil, but in time it shall force itself upon the notice of men, no longer an insignificant nut, but a great spreading tree under whose cooling shade, generations yet unborn may refresh their weary limbs.

